# MicroRNA miR-19b-3p mediated G protein γ subunit 7 (GNG7) loss contributes lung adenocarcinoma progression through activating Hedgehog signaling

**DOI:** 10.1080/21655979.2021.1976896

**Published:** 2021-10-11

**Authors:** Xia Zhao, Xiang-cheng Zhang, Kui Zang, Zhi-hao Yu

**Affiliations:** a Department of Ultrasound Medicine, The Affiliated Huaian No.1 People’s Hospital of Nanjing Medical University, Huaian, Jiangsu, PR China; b Department of Intensive Care Unit, The Affiliated Huaian No.1 People’s Hospital of Nanjing Medical University, Huaian, Jiangsu, PR China

**Keywords:** Human G protein gamma 7 (GNG7), miR-19b-3p, lung adenocarcinoma (LUAD), Hedgehog signaling

## Abstract

G protein γ subunit 7 (GNG7) is a subunit of heterotrimeric G protein. It has been demonstrated low expressed GNG7 in various cancers. Nevertheless, the role of GNG7 in lung adenocarcinoma (LUAD) remains unclear. In the present study, GNG7 expression in LUAD tissues and cell lines was analyzed by RT-qPCR, western blot and immunohistochemical. Kaplan–Meier analysis was performed for determining the prognostic value of GNG7 expression. Then, the function of GNG7 in LUAD progression was examined by cell proliferation, invasion and mouse xenograft assays. In addition, the underlying biological mechanisms of GNG7 in LUAD progression were explored via the bioinformatics analysis and experimental validation. We found GNG7 was markedly down-regulated in LUAD tissues and cell lines. Clinically, low expression of GNG7 was associated with the dismal prognosis of LUAD patients. Gain-of-function analysis showed that GNG7 overexpression inhibited proliferation and invasion of LUAD cell *in vitro*, and compromised tumor formation ability *in vivo*. Besides, mechanistic study revealed that overexpression of GNG7 affected the progression of LUAD via inhibiting activation of Hedgehog signaling. Moreover, bioinformatics prediction and experimental verification confirmed that GNG7 was targeted by miR-19b-3p, which was elevated expression in LUAD and promoting the progression of LUAD. Furthermore, rescue experiments demonstrated that GNG7 reintroduction weakened miR-19b-3p-mediated aggressive tumor phenotypes of LUAD cells. These findings suggested miR-19b-3p/GNG7 axis contributed to the progression of LUAD through Hedgehog signaling, which might be a potential therapeutic target for LUAD treatment.

## Introduction

Cancer are now widely recognized as a threat to global development [1,2]. Lung cancer, a prevalent respiratory malignancy, ranks the leading cause of cancer-related mortality worldwide [3]. Non-small cell lung cancer (NSCLC), mainly including adenocarcinoma (LUAD) and squamous cell carcinoma (LUSC), is the most common histological subtype of lung cancer [4]. And the incidence of lung adenocarcinoma is increasing yearly [5,6]. In recent years, surgery combined with immunotherapy and molecular targeted drugs greatly improving the prognosis of patients. However, the prognosis of advanced lung cancer is still not optimistic [7]. Therefore, searching for molecular markers and potential therapeutic targets for LUAD is still an important topic at present.

G protein γ subunit 7 (GNG7) is a subunit of heterotrimeric G protein. GNG7 plays a vital role in the process of neuro-protective response induced by A2A adenosine or D1 dopamine receptor [8]. Recent studies demonstrated that GNG7 is frequently down-regulated in various cancers, such as esophageal cancer, head and neck carcinoma, pancreatic cancer, and renal carcinoma [9–12]. In addition, there reported that GNG7 inhibited tumor progression through the mTOR pathway via inducing autophagy and cell death [13]. However, few studies showed the function and mechanisms of GNG7 during the initiation and progression of LUAD.

In this study, we showed that the expression of GNG7 was obviously lower in LUAD tissues than in normal tissues, and decreased GNG7 expression was associated with LUAD patients poor prognosis. GNG7 overexpression dampened NSCLC cell proliferation and invasion *in vitro*, and repressed tumorigenesis ability *in vivo*. Mechanistically, we also found that the overexpression of GNG7 affected LUAD progression through suppressing activation of Hedgehog signaling. In addition, we uncovered that GNG7 was target regulated by miR-19b-3p. Meanwhile, we confirmed miR-19b-3p was upregulated in LUAD tissue and promoted the progression of LUAD. In conclusion, our study demonstrated that overexpression of GNG7 inhibited the progression of LUAD through Hedgehog signaling, and miR-19b-3p was an upstream regulator of GNG7 in LUAD cells.

## Materials and method

### Sample collection and cell linse

Tissue microarray (TMA) containing LUAD tissues (n = 92) and normal tissues (n = 88) was purchased from Shanghai Core Super Biotechnology Co., Ltd (HLugA180Su04) The longest follow-up time for these patients is 5 years. Public DataBase (TCGA: The Cancer Genome Atlas; GEO: Gene expression omnibus) were downloaded and analyzed. The study protocols were approved by the research ethics committee of Nanjing Medical University. Human lung cancer cell lines (H460, A549, H1299, SK-MES-1, and NCI-H520) and normal bronchial epithelial cell line (16HBE) cells were purchased from ATCC (Manassas, USA) or Sibcb (Shanghai, China).

### Western blot

The protein isolation and western blotting were conducted according to the traditional protocol. Samples were probed with GNG7 (Abcam, USA; ab238868), SMO (Proteintech, China; 20,787-1-AP), GLI1 (Abcam, USA; ab134906), PTCH1 (Abcam, USA; ab53715) or GAPDH (Abcam, USA; ab9485) monoclonal antibody. Then, the samples were probed with Goat anti-mouse/anti-rabbit HRP antibodies. GAPDH was selected for internal controls.

### Quantitative real-time PCR (RT-qPCR)

Total RNA content was extracted using TRIzol reagent (Invitrogen, CA). The extracted RNA was then reversely transcribed into complementary DNA (cDNA) using a PrimeScript RT reagent Kit (Takara, Japan). Real-time quantitative polymerase-chain reaction (qPCR) was conducted using a Fast SYBR Green master mix (Applied Biosystems) on an ABI PRISM 7300 System (Applied Biosystemsin). The sequences of GNG7 primers were sense, 5ʹ- ATGTCAGCCACTAACAACATAGC-3ʹ; anti-sense, 5ʹ- AGACCTTGATGCGCTCAATCC-3ʹ. GAPDH primer sequences were sense, 5′-TGGTCACCAGGGCTGCTT-3′, and anti-sense, 5′-AGCTTCCCGTTCTCAGCC-3′. For detecting miR-19b-3p expression, the RT-qPCR reaction was performed using SYBR Premix Ex Taq kit (Takara, Japan). The sequences of miR-19b-3p primers were sense, 5′-ACACTCCAGCTGGGTGTGCAAATCCATGCAA-3′, and anti-sense 5′-CTCAACTGGTGTCGTGGAGTCGGCAATTCAGTTGAGTCAGTTTT-3′. U6 primers were sense 5′-CTTCGGCAGCACATATAC-3′, and anti-sense 5′-GAACGCTTCACGAATTTGC-3′. All the data of RT-qPCR were calculated and quantified using 2^−ΔΔCt^ method.

### Transfection of LUAD cell lines

Human-specific GNG7 cDNA clone were obtained from Shanghai Genechem Co., Ltd. MicroRNA mimics, inhibitor as well as respective negative controls (NC) of miR-29a-5p, miR-19b-3p, miR-708-5p, miR-20a-3p, miR-590-3p and miR-2355-5p were purchased from Gene Pharma (Shanghai, China).

### Luciferase activity assay

About 5 × 10^4^ A549 cells were seeded in a 24-well plate to reach 70% confluence. The wild type or miR-19b-3p binding site mutant 3ʹUTR of GNG7 were ligated into the pmirGLO luciferase reporter vector (Promega, WI), followed by co-transfection with control miRNAs or miR-19b-3p precursor lipofectamine 2000 for 48 hours. Luciferase activities were determined by a Dual‐ Luciferase Assay Kit (Promega, USA).

### In vivo tumorigenesis assay

The animal experiments were ratified by the Animal Ethics Committee of Nanjing Medical University. In brief, 10 female BALB/c nude mice (4–6 weeks; 18–20 g) (Vital River Laboratory, China) were randomly divided into two groups and then injected with A549 GNG7-OE cells or negative control to establish heterotopic subcutaneous xenotransplanted tumor model. Tumor volume was measured once a week and calculated based on the following equation: Volume = (length ×width^2^)/2.

### Cell Viability, Transmembrane invasion assay, EDU assay and Colony-forming assay

These assays were described in the Supplementary Materials and Methods.

### Statistical analysis

Data analysis was performed using SPSS 20.0 software (SPSS Inc., Chicago, IL). The data were shown as mean ± standard deviation. Student t-test was conducted for analyzing differences between the two groups, while one-way ANOVA was used for analyzing differences among more than two groups. The correlation of continuous variables was analyzed by Pearson correlation analysis. P < 0.05 was considered to be

a significant difference

## Result

### GNG7 was low expressed in LUAD

**Figure 1.**
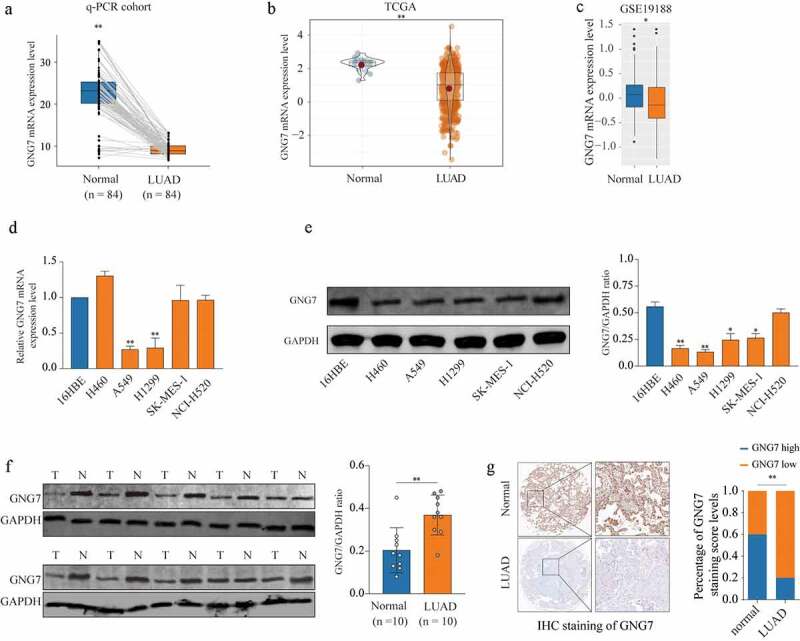
GNG7 was low expression in LUAD tissue and cell lines (a) mRNA level of GNG7 was detected in 84 pairs of LUAD tissues and normal tissues by RT-qPCR. (b-c) mRNA level of GNG7 in LUAD tissues and normal tissues from TCGA and GEO (GSE19188) database was compared. (d-e) mRNA and protein levels of GNG7 in Lung cancer cell lines (H460, A549, H1299, SK-MES-1 and NCI-H520) and Bronchial epithelial cell line(16HBE) were detected. (f) GNG7 protein expressions in 10 pairs of LUAD. The relative protein levels of GNG7 were normalized against GAPDH. (g) Tissue Microarray with LUAD tissues and normal tissues was used to detected expression of GNG7 by IHC, and the staining score of GNG7 percentage was quantified. * p < 0.05, **p < 0.005

Firstly, in order to explore whether GNG7 was dysregulated in LUAD, we determined the mRNA level of GNG7 in LUAD tissues and surrounding non-tumorous tissues through RT-qPCR. The result showed that GNG7 was frequently lower expressed in LUAD tissues (Figure 1a). And we found the results of the TCGA and GEO (GSE19188) databases were consistent with our findings (Figure 1b-c). Then, we detected mRNA and protein levels of GNG7 in multiple lung cancer cell lines and normal bronchial epithelial cell line (16HBE). We found both mRNA and protein levels of GNG7 in A549 and H1299 were lower then 16HBE (Figure 1d-e). Consistent with this, GNG7 protein was decreased in primary LUAD tumors tissues by western blot (n = 10) (figure 1f). To further demonstrate our findings, we detected the expression of GNG7 in tissue microarray (TMA) containing 92 LUAD tissues and 88 normal tissues through IHC staining, and the results showed that GNG7 level was indeed decreased in LUAD tissues (Figure 1g). These results indicated that GNG7 may exert tumor suppressive function in LUAD.

### Decreased GNG7 level was correlated with worse prognosis of LUAD patients

**Figure 2.**
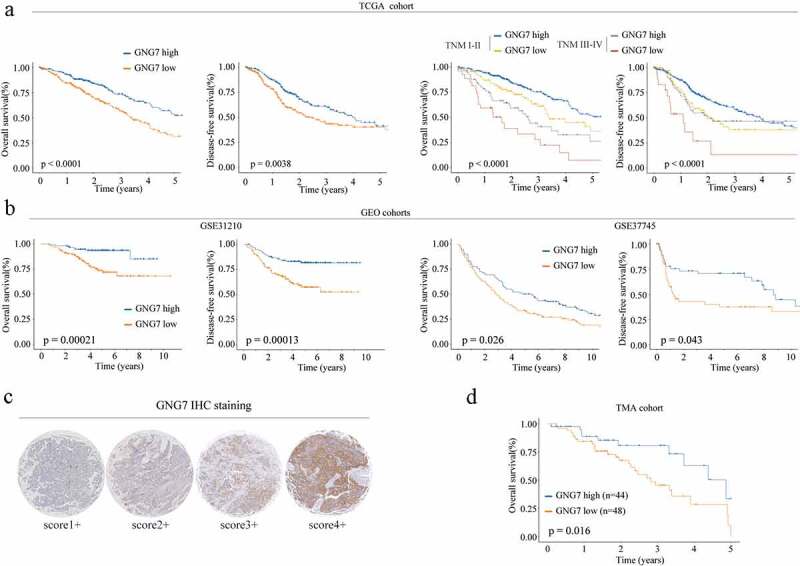
Low expression of GNG7 was correlated with poor prognosis of LUAD patient**s** (a-b) Relationship between GNG7 expression and prognosis of LUAD patients in TCGA and GEO cohorts. (c) IHC results of LUAD tissues and normal tissues were scored according to the degree of staining according to 1–4. (d) Kaplan–Meier analysis was used to analyze correlation between GNG7 expression and prognosis of LUAD patients

Then, we a explore the relationship between GNG7 level and clinical outcome of LUAD patients. TCGA dataset analysis showed that both overall survival time (OS) and disease-free survival (DFS) of patients with low GNG7 expression was significantly shorter than that of patients with high GNG7 expression (Figure 2a **left panel**). In addition, subgroup analysis based on the TNM stage (TNM I–II *vs*. III–IV) showed that low expression of GNG7 was also associated with worse OS and DFS rates in different TNM stages (Figure 2a **right panel**). Moreover, GSE database analysze results (GSE31210 and GSE37745) also confirmed that low expression of GNG7 was related to poor prognosis (Figure 2b). To further verify the reliability of the above results, we analyzed the relationship between GNG7 expression and prognosis of LUAD patients in the TMA cohort through IHC and similar result was observed (Figure 2c-d). These observations collectively indicate that the low expression of GNG7 is closely correlated with poor prognosis in NSCLC.

### Overexpression of GNG7 inhibits proliferation, migration and invasion of LUAD cells

**Figure 3.**
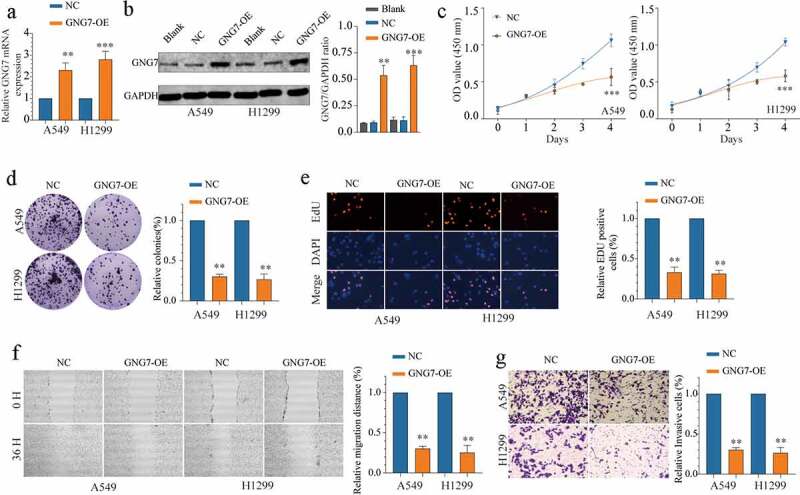
Overexpression of GNG7 inhibits proliferation, migration and invasion of LUAD cells (a-b) mRNA and protein levels of GNG7 were increased after transfection with GNG7-overexpressed plasmid in A549 and H1299 cells. (c) Cell viability of A549 and H1299 cells under treatment of GNG7 overexpressed plasmid and negative control in 1d,2d 3d and 4d. (d-e) GNG7 overexpression inhibited cell proliferation in LUAD cells, as demonstrated by colony formation and EDU assays, respectively. (f) The cell migration ability was measured by wound healing experiment in 0 h and 36 h after LUAD cell lines transfected with GNG7-overexpressed plasmid and negative control. (g) The invasion ability of A549 and H1299 cells with GNG7 overexpression were significantly suppressed according to cell invasion assay. Error bar represents the mean ± SD of three independent experiments. **p < 0.005.***p < 0.0005

To investigate the functional role of GNG7 in LUAD, we selected A549 and H1299 cell lines for our further study. First, we conducted stable cell lines with GNG7-overexpressed, and the transfection efficiency was verified both in mRNA and protein levels (Figure 3a,b). Then, we conducted CCK-8, colony formation and EDU experiments, and the results showed that overexpression of GNG7 significantly inhibited cell proliferation ability of LUAD cells (Figure 3c-e). In addition, to investigate the relationship between GNG7 expression and metastasis abilities of LUAD cells, we conducted wound healing experiment and transwell invasion assay. Noticeably, overexpression of GNG7 could suppress migration and invasion abilities of LUAD cells (figure 3f-g). These *in vitro* data reveal that GNG7 impeded tumor proliferation and metastasis.

### Overexpression of GNG7 inhibits tumorigenesis ability

**Figure 4.**
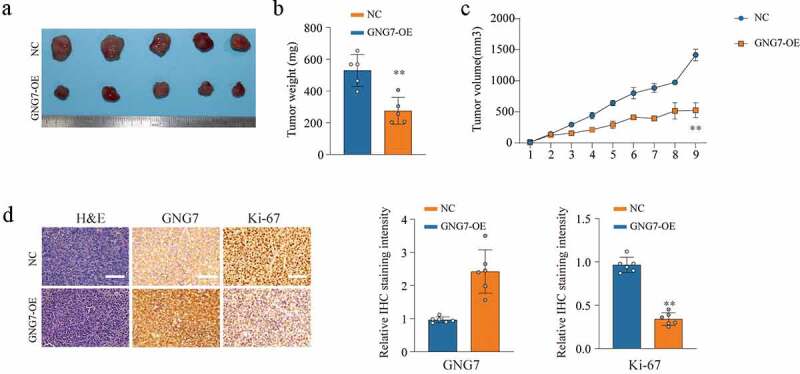
Overexpression of GNG7 inhibits tumorigenesis ability of LUAD cells *in vivo* (a-c) The *in vivo* effect of GNG7 was evaluated in xenograft mouse models bearing tumors originating from A549 cells via tumor volume and tumor weight; n = 5 per group. (d) Expression of GNG7 and Ki67 were detected through IHC staining between GNG7 overexpression and negative control groups in node mouse tumor tissues. **p < 0.005.***p < 0.0005

To further investigate the role of GNG7 in tumorigenesis ability *in vivo*, we used stable cell lines with GNG7-overexpressed and negative control to conduct mice xenograft tumor model. As exhibited in (Figure 4a-c), tumor weight and tumor volume were observably lower and smaller in the GNG7-overexpressed group than that in control group (Figure 4a-c). In addition, the results of IHC showed that the Ki-67 level in the GNG7-overexpressed group was lower than the negative control group (Figure 4d). These results indicated that GNG7 restrained LUAD growth *in vivo*.

### GNG7 is a direct target of miR-19b-3p in LUAD cells

**Figure 5.**
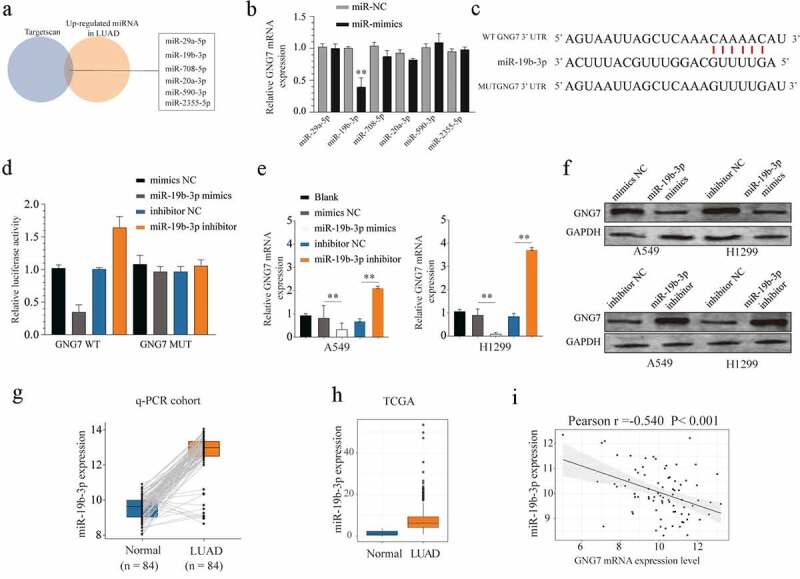
GNG7 was target regulated by miR-19b-3p in LUAD cells (a-c) Candidate miRNAs which upregulated in LUAD tissues and were complementary pairing with 3ʹUTR of GNG7 were showed, and GNG7 mRNA level was detected under condition of the candidate miRNAs mimics and negative control. (d) Relative luciferase activity between GNG7 wild type and mutant type based on a dual-luciferase reporter assay. (e, f) Protein and mRNA change of GNG7 in A549 and H1299 cells under treatment of miR-19b-3p mimics, inhibitor and negative control. (g-h) Expression difference of miR-19b-3p between LUAD tissues and normal tissues in RT-qPCR cohort and TCGA cohort. (mi) relationship between GNG7 expression and miR-19b-3p expression in LUAD tissues. Error bar represents the mean ± SD of three independent experiments. **p < 0.005.***p < 0.0005

To explore the upstream molecular mechanism of GNG7 dysregulation, the potential miRNAs targeting GNG7 were predicted by bioinformatics database (Targetscan). We selected the candidate miRNAs that were upregulated in LUAD tissues. Then, we used Targetscan to identify those miRNAs with complementary pairing with 3ʹUTR of GNG7. We found miR-29a-5p, miR-19b-3p, miR-708-5p, miR-20a-3p, miR-590-3p and miR-2355-5p were available for our study (Figure 5a). However, only miR-19b-3p mimics could significantly decrease the mRNA level of GNG7 in LUAD cell lines (Figure 5b-c). We also conducted a luciferase experiment to demonstrate that miR-19b-3p could be specifically binding to the 3ʹUTR sequence of GNG7 in LUAD cell lines (Figure 5d). In addition, we demonstrated that miR-19b-3p mimics greatly decreased the mRNA and protein level of GNG7 in LUAD cell lines, while miR-19b-3p silencing could display the opposite result (Figure 5e-f). Then, we demonstrated that miR-19b-3p was overexpressed in LUAD tissue using the RT-qPCR cohort (Figure 5g) and TCGA database (Figure 5h). Moreover, correlation analysis showed expression of miR-19b-3p was negatively correlated with the mRNA level of GNG7 in LUAD tissue (Figure 5i). Taken together, these findings indicated that GNG7 is a direct downstream target of miR-19b-3p.

### Overexpression of GNG7 could counteract the tumor-promoting effect mediated by miR-19b-3p

**Figure 6.**
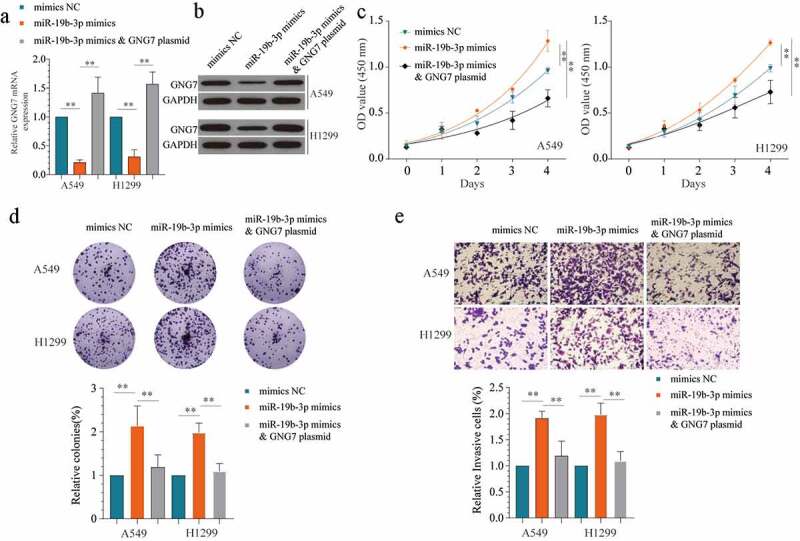
Overexpression of GNG7 could reverse the tumor-promoting effect of miR-19b-3p (a, b) showed GNG7 mRNA and protein expression under condition of miR-19b-3p mimics, miR-19b-3p mimics & GNG7 plasmid or negative control. (c) Cell viability of A549 and H1299 cells under treatment of miR-19b-3p mimics, miR-19b-3p mimics & GNG7 plasmid and negative control in 1d, 2d, 3d and 4d. (d) Colony formation ability of A549 and H1299 cells under treatment of miR-19b-3p mimics, miR-19b-3p mimics & GNG7 plasmid and negative control was compared in vitro, and the result was quantified. (e) Cell invasion ability of A549 and H1299 cells under treatment of miR-19b-3p mimics, miR-19b-3p mimics & GNG7 plasmid and negative control was detected by transwell assay, and the result was quantified. Error bar represents the mean ± SD of three independent experiments. **p < 0.005.***p < 0.0005, unpaired Student’s t test or one-way ANNOVA followed by multiple t test

To further verify the relationship between GNG7 and miR-19b-3p on the progression of LUAD, we used miR-19b-3p mimics, miR-19b-3p mimics & GNG7 plasmid and negative control to treatment LUAD cell lines. The transfect efficiency was verified by detecting the mRNA and protein level of GNG7 (Figure 6a,b). Then, the results of CCK-8 and colony formation showed that overexpression of GNG7 could reverse the proliferation -promoting effect of miR-19b-3p (Figure 6c-d). Similarly, in transwell assays, miR-19b-3p mimics increased LUAD cell invasion ability, whereas GNG7 reintroduction blocked this change (Figure 6e). Hence, our findings demonstrated that miR-19b-3p regulates LUAD cell proliferation and invasion via targeting GNG7.

### miR-19b-3p/GNG7 axis regulates the progression of LUAD through Hedgehog signaling

**Figure 7.**
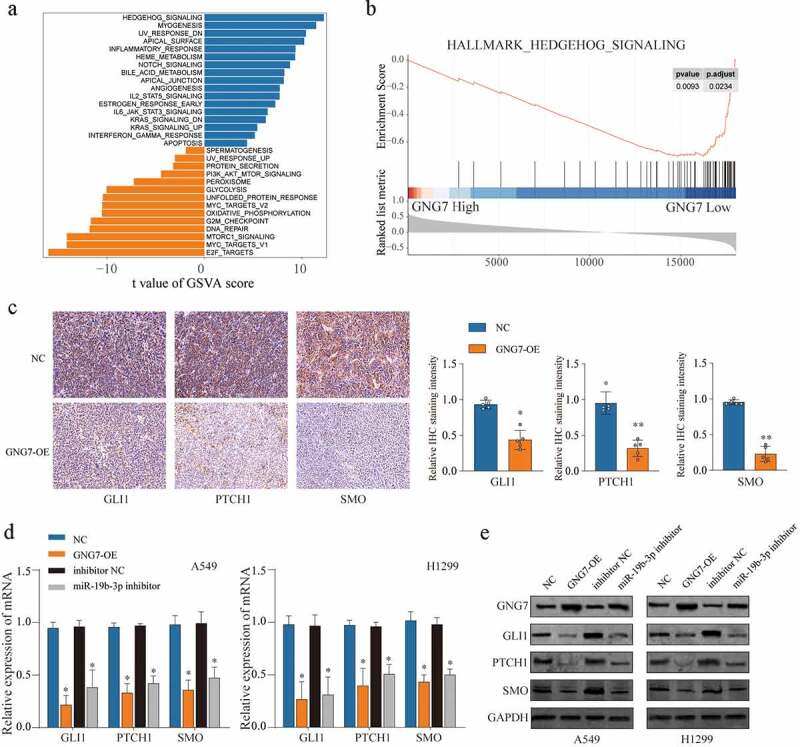
miR-19b-3p/GNG7 axis regulates progression of LUAD through hedgehog signaling the gene set enrichment analysis (a) and the gene set variation t analysis (b) was conducted in TCGA LUAD cohort between GNG7 high expression and GNG7 low expression, and indicated a significant correlation between GNG7 and Hedgehog pathway. (c) IHC staining for the change of SMO, GLI1 and PTCH1 levels after GNG7 overexpression. (d-e) Expression change of mRNA and protein levels of SMO, GLI1 and PTCH1 in A549 and H1299 cells which transfected with GNG7 overexpression plasmid or miR-19b-3p inhibitor, respectively. Error bar represents the mean ± SD of three independent experiments. **p < 0.0005

To investigate the mechanism that GNG7 affects the aggressive phenotype of LUAD cells, we searched the relevant pathways which activation was associated with the expression of GNG7 in LUAD and found Hedgehog signaling was the most significant correlation with expression of GNG7 through Gene set variation analysis (GSVA) (Figure 7a) and Gene Set Enrichment Analysis (GSEA) (Figure 7b). Furthermore, IHC assays revealed that GNG7 overexpression resulted in distinct suppression of Hedgehog signaling pathway-related molecules (GLI1, PTHCH1 and SMO) in tumor tissues obtain from xenograft tumor model (Figure 7c). Then, we detected Hedgehog signaling-related proteins’ expression under the condition of GNG7 overexpression or miR-19b-3p inhibition, respectively, in LUAD cell lines. The results showed that both mRNA and protein levels of SMO, GLI1 and PTCH1 were decreased following GNG7 overexpression or miR-19b-3p inhibition (Figure 7d). Overall, our findings suggested miR-19b-3p/GNG7 axis regulates LUAD progression via modulating Hedgehog signaling.

## Discussion

GNG7 downregulation has been observed in various cancers, and GNG7 acted as a tumor-suppressor gene involved in cancer progression. For instance, M Ohta1 *et al*. found that e GNG7 was frequently suppressed expression in esophageal cancer, and low GNG7 expression was associated with the metastatic potential of esophageal cancer cells and poorer prognosis in the clinical patient cohort [12]. Hartmann *et al*. reported that a loss of GNG7 protein expression is observed in head and neck tumor [11]. Xu *et al*. proved that GNG7 was downregulated in clear cell renal cell carcinoma tissues and function as a cancer suppressor protein [9]. Consistently, in this study, we proved GNG7 was down-regulated in LUAD tissue. Low expression of GNG7 correlated with poor prognosis of LUAD patients using multiple cohorts both in mRNA and protein level.

Subsequently, functional experiments were conducted to investigate the role of GNG7 in LUAD. The results showed that overexpression of GNG7 remarkedly attenuated cell proliferation and invasion *in vitro* and suppressed tumorigenesis ability *in vivo*. These results were in accordance with the findings observed in clear cell renal cell carcinoma, which also proven that GNG7 function as a tumor suppressor gene [9]. Moreover, Zheng *et al.* proved that GNG7 could suppress the progression of lung adenocarcinoma by inhibiting E2F transcription factor 1 [14]. The above results indicated that loss of GNG7 expression might play a critical role in NSCLC progression. However, the underlying mechanisms related to the GNG7 function role remain unknown.

To further investigate the signals related to GNG7, we searched the signaling pathway changes under different levels of GNG7 in LUAD tissue and found Hedgehog signaling displayed the most remarkable change. Recent studies have shown that the Hedgehog signaling pathway was closely related to tumor proliferation, invasion and metastasis, angiogenesis, drug resistance and apoptosis [15]. Many research showed that activation of Hedgehog signaling pathway was an important factor for the development of lung cancer. Szczepny *et al*. demonstrated that activation of Hedgehog signaling pathway was important for the progression of small cell lung cancer (SCLC). Silencing Shh obviously suppressed the tumor growth, whereas the overexpression of Shh leads to larger tumors in mice model [16]. In NSCLC, the Hedgehog signaling pathway played as critical role during tobacco-induced oncogenesis [17]. Of note, our results indicate that GNG7 overexpression suppressed Hedgehog signaling related proteins expression, which indicating GNG7 suppressed the progression through the modulation of Hedgehog signaling pathway.

MiRNAs are non-coding RNAs with ~22 nucleotides which affect gene expression via suppressing post-transcriptional translation of target mRNAs [18]. To further investigate the mechanism of GNG7 loss in LUAD cells, those miRNAs which both were upregulating in LUAD tissues and complementary pairing with 3ʹUTR of GNG7 were selected, then we selected miR-19b-3p for further validation . Further experiments showed that only the miR-19b-3p was meeting the requirements of our study. miR-19b-3p has been identified as a tumor promoter in several types of cancer, including esophageal cancer, intrahepatic cholangiocarcinoma, and lung cancer [19–21]. Additionally, a recent study has shown that miR-19b-3p was overexpressed in NSCLC cell lines and acted as a tumor-promoter during the progression of NSCLC [22,23]. Consistently, we demonstrated that miR-19b-3p could be specifically binding to the 3ʹUTR sequence of GNG7 in LUAD cell lines. Rescue experiment proven that enforced expression of GNG7 could partial abrogated the tumor-promoting effect caused by miR-19b-3p. Moreover, GNG7 overexpression or miR-19b-3p inhibition both restrained Hedgehog signaling pathway activation. Collectively, miR-19b-3p accelerated LUAD progression by GNG7 mediated Hedgehog signaling pathway in-activation.

## Conclusions

In conclusion, our study confirmed that GNG7 is frequently down-regulated in LUAD and associated with a poor prognosis of LUAD patients. Overexpression of GNG7 inhibit proliferation, metastasis and tumor formation ability of LUAD cells via in-activation Hedgehog pathway. Further study shows that the mechanism of GNG7 loss might partially due to miR-19b-3p dysregulated expression, which could induce the degradation of GNG7 mRNA in LUAD cells. Based on these results, GNG7 may become a potential target for targeted therapy of LUAD.

## Supplementary Material

Supplemental Material
