# Supplementary material for: MicroRNA miR-19b-3p mediated G protein γ subunit 7 (GNG7) loss contributes lung adenocarcinoma progression through activating Hedgehog signaling
Source: Bioengineered. 2021 Oct 11;12(1):7849–58. doi: 10.1080/21655979.2021.1976896 (PMC8806737; doi:10.1080/21655979.2021.1976896)

**Supplementary materials**

*Cell Viability*

After indicated transfection, these cells were seeded into 96-well microplates with 5,000 cells per well to perform the cell viability with Cell counting kit-8 (CCK-8) assay (Dojindo, Kumamoto, Japan) in 1d, 2d, 3d and 4d. The OD450 value was determined by using a MRX II microplate reader (Dynex, USA).

*Transmembrane invasion assay*

After indicated transfection, the transmembrane invasion experiment was performed according to the introduction of the Transwell system (Corning, USA). After 24 h incubation, the cells that invaded through the membrane were fixed with methanol and stained with 0.5% crystal violet.

*EDU assay*

After indicated transfection, 10 μM EdU for 2h before fixation, permeabilization, and EdU staining. Cell nuclei were stained with Hoechst 33342 (Invitrogen) at a concentration of 5 μg/mL for 30 min.

*Colony-forming assay*

After indicated transfection, cells were dissociated with trypsin, resuspended in DMEM complete medium with 10% FBS, and inoculated into a 6-well plate at a density of 2000 cells/well. After 12 days, colonies were washed twice with PBS, dyed with crystal violet and photographed.

Figure S1 panoramic scanning for LUAD TMA cohort

Figure S2 Relationship between expression of GNG7 and TNM stage of LUAD patients from GEPIA online tools (http://gepia.cancer-pku.cn/)


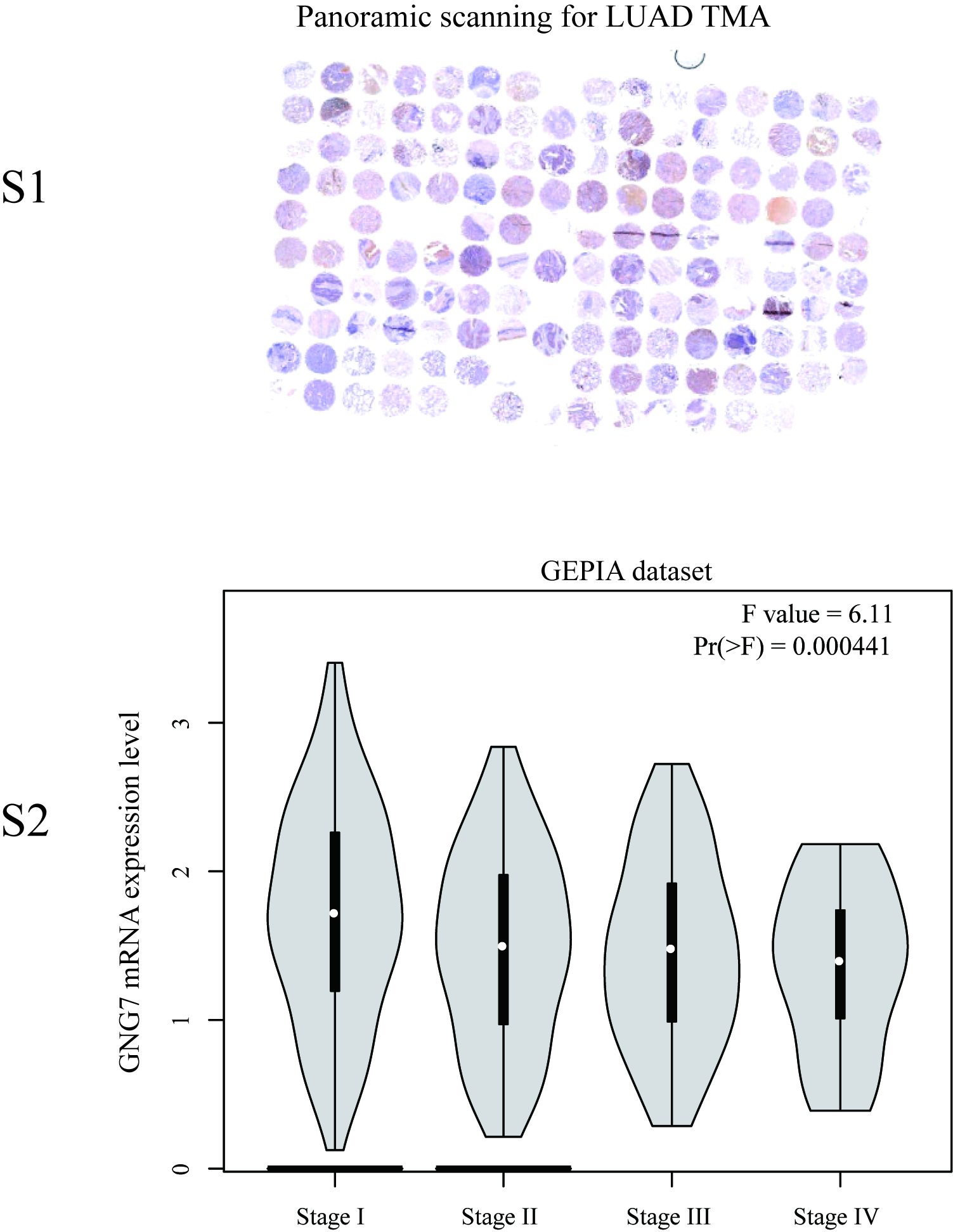

Supplement: Supplemental Material [file KBIE_A_1976896_SM8137.zip › supplementary/Supplementary materials.docx]
