# Supplementary figures and images for: MicroRNA miR-19b-3p mediated G protein γ subunit 7 (GNG7) loss contributes lung adenocarcinoma progression through activating Hedgehog signaling
Source: Bioengineered. 2021 Oct 11;12(1):7849–58. doi: 10.1080/21655979.2021.1976896 (PMC8806737; doi:10.1080/21655979.2021.1976896)

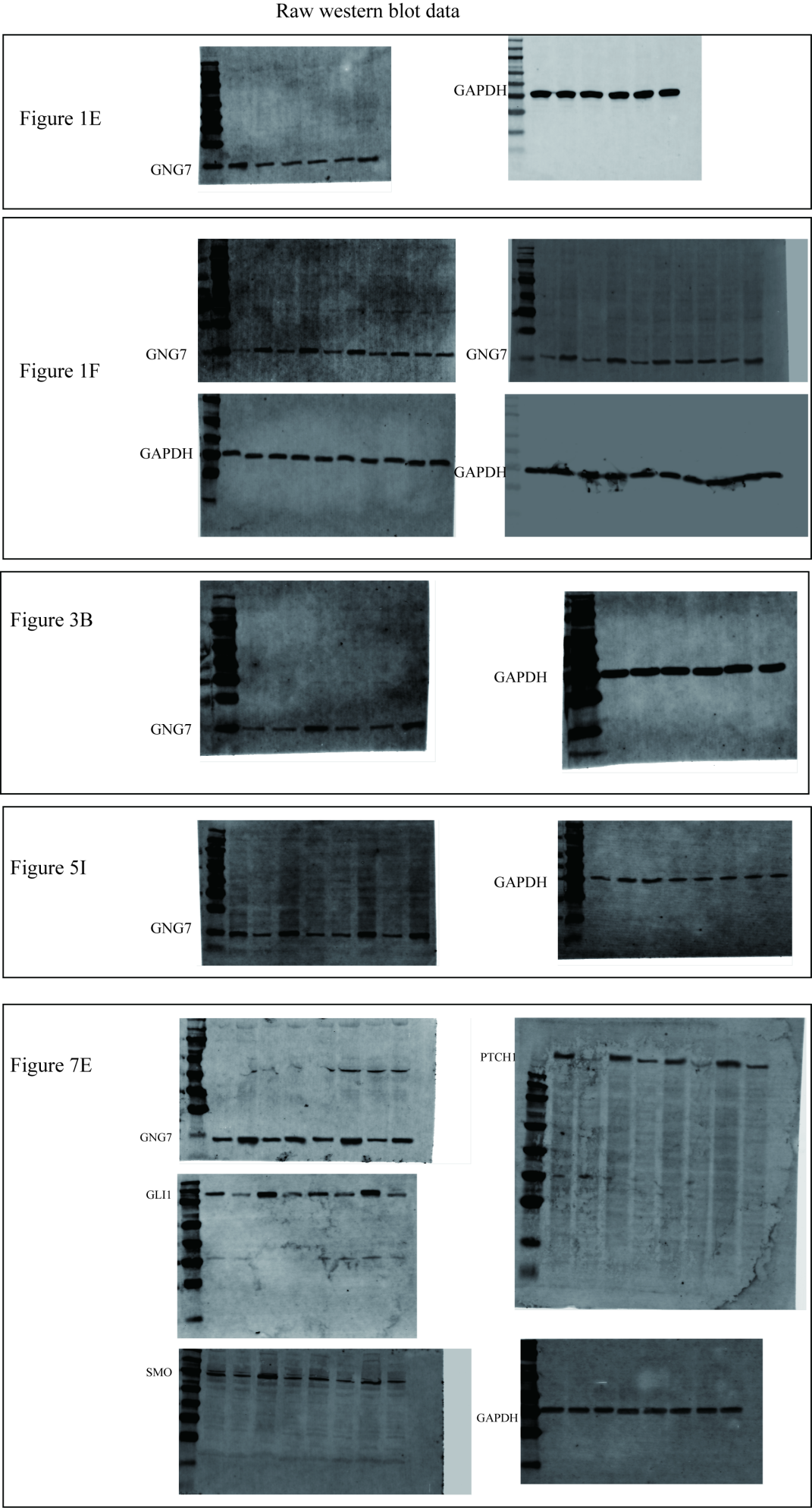

Supplement: Supplemental Material [file KBIE_A_1976896_SM8137.zip › supplementary/Raw western blot data.docx]
